# Factors impacting cumulative dissipated energy levels and postoperative visual acuity outcome in cataract surgery

**DOI:** 10.1186/s12886-021-02205-w

**Published:** 2021-12-20

**Authors:** Anh D. Bui, Zhimin Sun, Yunzhen Wang, Shengsong Huang, Michael Ryan, Yinxi Yu, Gui-Shuang Ying, Saras Ramanathan, Kuldev Singh, Yangfan Yang, Ying Han

**Affiliations:** 1 Department of Ophthalmology, University of California, San Francisco, 490 Illinois Street, San Francisco, CA 94158 USA; 2 State Key Laboratory of Ophthalmology, Zhongshan Ophthalmic Center, Sun Yat-sen University, Guangzhou, China; 3 Department of Ophthalmology, University of Pennsylvania, Philadelphia, PA USA; 4 Department of Ophthalmology, Stanford University, Stanford, CA USA

**Keywords:** Cataract, Phacoemulsification, Cumulative dissipated energy, CDE, Visual outcome

## Abstract

**Purpose:**

To determine factors impacting cumulative dissipated energy (CDE) and postoperative best-corrected visual acuity (BCVA) in phacoemulsification.

**Design:**

Review of 1102 cases at University of California, San Francisco (UCSF) and at Zhongshan Ophthalmic Center (ZOC), China.

**Subjects:**

Patients who underwent cataract surgery at UCSF 03/2014–03/2019 and at ZOC 10/2018–05/2019.

**Methods:**

Patient demographics, medical history, routine ocular examination, and surgical information, including disassembly method, complications, and surgeon training level were recorded. Univariable and multivariable regression models were used to determine factors associated with CDE and good postoperative BCVA (20/40 or better) at 1 month.

**Outcome measures:**

CDE, postoperative BCVA.

**Results:**

In multivariable analysis, patient age at time of surgery, diabetes, degree of nuclear sclerosis (NS), white-to-white corneal diameter, disassembly method, preoperative BCVA, surgeon training level, and surgical center were significantly associated with CDE. Log_10_CDE increased by 0.20–0.31 for patient age ≥ 70 years, by 0.07 if the patient had diabetes, by 0.12–0.41 for NS grade ≥ 2, by 0.48 per 10 mm increase in white-to-white corneal diameter, by 0.34–0.47 for disassembly method other than non-stop chop, by 0.16 per unit increase in preoperative logMAR BCVA, and by > 0.09 when phacoemulsification was performed by residents early in their training. Log_10_CDE was 0.33 higher at UCSF than ZOC. In multivariable analysis, worse baseline visual acuity and age above 90 years at time of surgery decreased the odds of good BCVA (OR = 0.26 per unit increase in preoperative logMAR BCVA; OR = 0.12 for age > 90); comorbid retinal issues decreased the odds of good postoperative BCVA (OR = 0.13–0.39); greater anterior chamber depth (ACD) or shorter axial length (AL), increased the odds of good postoperative outcome (OR = 2.64 per 1 mm increase ACD, OR = 0.84 per 1 mm increase AL).

**Conclusions:**

Cataract grade determined by slit lamp exam and, for the first time, older patient age, were noted to be important predictors of high CDE. CDE was not a risk factor for postoperative BCVA measured at postoperative 1 month. When surgery was performed by trainees under supervision, lower training level was associated with higher CDE, but not with worse postoperative BCVA.

**Supplementary Information:**

The online version contains supplementary material available at 10.1186/s12886-021-02205-w.

Cataract surgery is the most commonly performed ophthalmic surgery. Phacoemulsification has become the method of choice for lens removal in uncomplicated cataracts. The outcomes of phacoemulsification surgery for vision restoration are excellent [1]. The present goals for improvements in phacoemulsification methods are thus focused on optimizing refractive results, accelerating recovery speed, and reducing complication rates.

Two key objectives during phacoemulsification are to: 1) avoid or minimize any trauma to nearby ocular issue and 2) maximize the visual acuity after phacoemulsification. Besides direct instrumental damage to ocular tissues, the degree of ultrasonic exposure during phacoemulsification is a hidden but important determinant of iatrogenic injury, such as corneal endothelial cell loss, which may lead to long-term complications. Ultrasonic exposure results in heat dissipation within the corneal tissues and may cause acoustic cavitation and hydroxyl radical generation within the anterior chamber [2, 3]. While decline of endothelial cell count is expected with advancing age, accelerated rates of endothelial cell loss have been reported after surgery, ranging from ~ 4–20% within the year following cataract surgery [4–7]. The amount of ultrasonic exposure during phacoemulsification is typically quantified using cumulative dissipated energy (CDE), which is measured and reported by the phacoemulsification systems. The second outcome is visual acuity after phacoemulsification, which is the primary goal of cataract surgery in majority of cases.

Despite the fact that several studies looked at factors predictive for high CDE levels and final visual acuity [6, 8], our understanding of the patient-specific and surgery-related factors that contribute to the variation of intraoperative CDE and final visual acuity remains limited, especially for trainees’ performed phacoemulsification. Limitations from previous studies include that cohort sizes were typically small and some commonly encountered factors (such as age at time of surgery) were not analyzed, potentially limiting the generalizability of the study findings [6, 8]. Furthermore, some factors that have been analyzed are not always easily obtainable clinically. In particular, while it has been well-established that lens density affects CDE, this association has mainly been studied by slit-lamp images with standard photographic charts of the Lens Opacities Classification System III (LOCS III) system or a Scheimpflug imaging system (Pentacam), neither system is commonly used in daily practice. The relationship between CDE and clinical grading of lens density during slit lamp examination has not been studied. Finally, the association between CDE and postoperative outcomes is also limited.

The purpose of this study is to investigate a broad set of risk factors that affect CDE levels and postoperative BCVA in a large cohort of patients who had phacoemulsification surgery at two large and independent teaching centers. Identification of factors associated with CDE and visual acuity may improve teaching of phacoemulsification surgery, guide surgeons in optimizing phacoemulsification and better prepare for difficult cases to improve surgical outcomes.

## Methods

### Study population

This study was conducted in compliance with the rules and regulations of the Health Insurance Portability and Accountability Act and all applicable federal and state laws, and in adherence to the tenets of the Declaration of Helsinki. The study was approved by the Institutional Review Board at the University of California, San Francisco (UCSF) and Zhongshan Ophthalmic Center (ZOC) in Guangzhou, China.

This study included patients who met the inclusion criteria and underwent phacoemulsification and intraocular lens implantation at UCSF between March 1, 2014 and March 1, 2019. Data from patients who underwent phacoemulsification and intraocular lens implantation at ZOC from October 1, 2018 to May 1, 2019 was accumulated. The inclusion criteria included: (1) ability to identify primary surgeon, (2) adult patients (age > 18 years). The exclusion criteria included: (1) missing CDE data, (2) combined cases, such as combined corneal transplants, glaucoma surgeries, or retinal surgeries. Corneal or retinal conditions, glaucoma, or uveitis were not criteria for exclusion and were included as risk factors in the analysis. Demographic and clinical information was recorded, including age, gender, race, medical and surgical history, medications, allergies, and family history of ocular disease.

### Cataract grading

Cataract grading was performed by the surgeon on clinical slit lamp examination during the preoperative evaluation. A modified Lens Opacities Classification System III (LOCS III) was used to grade the cataracts in the ophthalmology clinic at both UCSF and ZOC [9]. Observers were attending physicians who were fully trained on the classification system. For nuclear cataract, the range was from grade 1 for mild nuclear opacification and color to grade 4 for severe nuclear opacification. Cortical cataract was graded by degree of intrapupillary space obscured, with less than 10% for grade 1, 10–50% for grade 2, 50–90% for grade 3, and more than 90% for grade 4. Posterior subcapsular cataract was graded by degree of posterior capsule obscured, with less than 3% for grade 1, 3–30% for grade 2, 30–50% for grade 3, and more than 50% for grade 4.

### Cataract surgical procedure

Phacoemulsification was performed in standard fashion under topical or sub-Tenon’s anesthesia (Centurion; Alcon Laboratories, Inc). Briefly, after a temporal clear corneal incision was created, an anterior capsulorhexis was performed, followed by hydrodissection with or without hydrodelineation. The lens nucleus was removed utilizing non-stop chop, stop and chop, or divide and conquer. Both centers used the same strategy to optimize the phacoemulsification machine setting parameters. An acrylic intraocular lens (IOL) was inserted into the bag after removing the epinucleus and cortex when no complications occurred. Otherwise, the IOL was placed into the sulcus or anterior chamber or the patient was left aphakic, and complications (e.g., posterior capsule rupture with or without vitrectomy, dropped lens, and zonular dialysis) were noted for inclusion in the analysis as a risk factor. Prophylactic intracameral or subconjunctival antibiotic was given.

### Outcome measures and analyzed factors

CDE, recorded from the phacoemulsification system, was analyzed as a continuous response variable. Preoperative and postoperative Snellen best-corrected visual acuity (BCVA) were measured on the logarithm of the minimum angle of resolution (logMAR) scale. Postoperative BCVA was transformed into a dichotomous outcome, with logMAR ≤0.3 (Snellen equivalent 20/40) considered as good postoperative BCVA and logMAR > 0.3 considered as poor postoperative BCVA. Postoperative BCVA was measured with manifest refraction approximately 1 month after cataract surgery. Twenty-one risk factors (Table 1) were recorded and analyzed for association with these response variables (e.g., CDE and BCVA). These risk factors included cataract grade (see “Cataract Grading”), preoperative BCVA and postoperative BCVA, and other factors grouped according to the following categories: patient demographics, patient ocular geometry, patient medical history, surgical method, and surgeon training and affiliation. Complications (e.g., capsular rupture with or without vitrectomy, dropped lens, zonular dialysis) were also documented.

**Table 1:** Baseline characteristics of study participants in UCSF cohort and ZOC cohort

	UCSF (*N* = 864 eyes)	ZOC (*N* = 238 eyes)	Combined (*N* = 1102 eyes)
**Patient-Level Characteristics**			
**Age at the time of surgery (years)**			
Mean (SD)	72.1 (9.8)	65.7 (11.8)	70.7 (10.6)
< 60	82 (9.5%)	55 (23.1%)	137 (12.4%)
60–69	258 (29.9%)	91 (38.2%)	349 (31.7%)
70–79	354 (41.0%)	70 (29.4%)	424 (38.5%)
80–89	141 (16.3%)	22 (9.2%)	163 (14.8%)
> =90	29 (3.4%)	0 (0.0%)	29 (2.6%)
**Gender**			
Male	327 (37.8%)	109 (45.8%)	436 (39.6%)
Female	537 (62.2%)	129 (54.2%)	666 (60.4%)
**Diabetics: Yes (%)**	213 (24.7%)	45 (18.9%)	258 (23.4%)
**Eye-Level Characteristics**			
**Uveitis comorbid: Yes (%)**	6 (0.7%)	2 (0.8%)	8 (0.7%)
**Cornea comorbid**			
No	862 (99.8%)	234 (98.3%)	1096 (99.5%)
Scarred or cloudy cornea	1 (0.1%)	4 (1.7%)	5 (0.5%)
Penetrating keratoplasty/endothelial keratoplasty	1 (0.1%)	0 (0.0%)	1 (0.1%)
**Glaucoma comorbid**			
No	622 (72.0%)	212 (89.1%)	834 (75.7%)
Incisional surgery	12 (1.4%)	12 (5.0%)	24 (2.2%)
Pseudoexfoliation glaucoma	24 (2.8%)	0 (0.0%)	24 (2.2%)
Primary angle closure disease	43 (5.0%)	14 (5.9%)	57 (5.2%)
Other	163 (18.9%)	0 (0.0%)	163 (14.8%)
**Retina comorbid**			
None	824 (95.4%)	215 (90.3%)	1039 (94.3%)
Pars plana vitrectomy	4 (0.5%)	23 (9.7%)	27 (2.5%)
Other	36 (4.2%)	0 (0.0%)	36 (3.3%)
**Nuclear sclerotic cataract grade**			
Unknown	6 (0.7%)	0 (0.0%)	6 (0.5%)
0	23 (2.7%)	0 (0.0%)	23 (2.1%)
1	160 (18.5%)	17 (7.1%)	177 (16.1%)
2	471 (54.5%)	49 (20.6%)	520 (47.2%)
3	192 (22.2%)	139 (58.4%)	331 (30.0%)
4	12 (1.4%)	33 (13.9%)	45 (4.1%)
**Cortical cataract grade**			
Missing	3 (0.3%)	0 (0.0%)	3 (0.3%)
0	565 (65.4%)	13 (5.5%)	578 (52.5%)
1	91 (10.5%)	115 (48.3%)	206 (18.7%)
2	145 (16.8%)	63 (26.5%)	208 (18.9%)
3	54 (6.3%)	45 (18.9%)	99 (9.0%)
4	6 (0.7%)	2 (0.8%)	8 (0.7%)
**Posterior subcapsular cataract grade**			
Missing	4 (0.5%)	0 (0.0%)	4 (0.4%)
0	721 (83.4%)	0 (0.0%)	721 (65.4%)
1	51 (5.9%)	140 (58.8%)	191 (17.3%)
2	37 (4.3%)	45 (18.9%)	82 (7.4%)
3	42 (4.9%)	28 (11.8%)	70 (6.4%)
4	9 (1.0%)	25 (10.5%)	34 (3.1%)
**Axial length (mm)**			
N	859	235	1094
Mean (SD)	24.1 (1.6)	24.3 (2.2)	24.1 (1.7)
**Anterior chamber depth (mm)**			
N	845	235	1080
Mean (SD)	3.1 (0.5)	3.1 (0.5)	3.1 (0.5)
**White-to-white corneal diameter (mm)**			
N	858	234	1092
Mean (SD)	11.8 (0.5)	11.7 (0.7)	11.7 (0.6)
**Pre-surgery logMAR visual acuity**			
N	864	238	1102
Mean (SD)	0.5 (0.4)	1.1 (0.5)	0.6 (0.5)
**Surgery-Level Characteristics**			
**Surgeon training level**			
Second year resident	2 (0.2%)	0 (0.0%)	2 (0.2%)
Early third year resident	192 (22.2%)	0 (0.0%)	192 (17.4%)
Late third year resident	236 (27.3%)	0 (0.0%)	236 (21.4%)
Fellow	246 (28.5%)	0 (0.0%)	246 (22.3%)
Attending	188 (21.8%)	238 (100.0%)	426 (38.7%)
**Disassembly method**			
Unknown	1 (0.1%)	0 (0.0%)	1 (0.1%)
Divide and conquer	20 (2.3%)	6 (2.5%)	26 (2.4%)
Stop and chop	135 (15.6%)	232 (97.5%)	367 (33.3%)
Non-stop chop	705 (81.6%)	0 (0.0%)	705 (64.0%)
2 handed technique	2 (0.2%)	0 (0.0%)	2 (0.2%)
Using the epinuclear setting	1 (0.1%)	0 (0.0%)	1 (0.1%)
**Pupil expansion or capsular support devices**			
None	835 (96.6%)	236 (99.2%)	1071 (97.2%)
Iris hooks	4 (0.5%)	1 (0.4%)	5 (0.5%)
Mackool hooks	1 (0.1%)	0 (0.0%)	1 (0.1%)
Capsular tension ring	2 (0.2%)	1 (0.4%)	3 (0.3%)
Mackool + Capsular tension ring	1 (0.1%)	0 (0.0%)	1 (0.1%)
Malyugen ring	20 (2.3%)	0 (0.0%)	20 (1.8%)
I-Ring	1 (0.1%)	0 (0.0%)	1 (0.1%)
**Loose zonules**	6 (0.7%)	6 (2.5%)	12 (1.1%)
**Complication status**			
None	845 (97.8%)	236 (99.2%)	1081 (98.1%)
Capsular rupture without vitrectomy	5 (0.6%)	0 (0.0%)	5 (0.5%)
Capsular rupture with vitrectomy	10 (1.2%)	0 (0.0%)	10 (0.9%)
Dropped lens	0 (0.0%)	2 (0.8%)	2 (0.2%)
Zonular dialysis, vitrectomy	1 (0.1%)	0 (0.0%)	1 (0.1%)
Other	3 (0.3%)	0 (0.0%)	3 (0.3%)
**CDE (Percent-seconds)**			
N	864	234	1098
Mean (SD)	9.4 (13.0)	11.1 (9.9)	9.8 (12.5)
Median	5.3	8.3	6.0
Q1, Q3	2.6, 10.6	4.5, 13.3	2.9, 11.5
Range	(0.0–119.0)	(0.2–55.3)	(0.0–119.0)
**Log10 (CDE+ 1)**			
N	864	234	1098
Mean (SD)	0.8 (0.4)	1.0 (0.3)	0.9 (0.4)
Median	0.8	1.0	0.8
Q1, Q3	0.6, 1.1	0.7, 1.2	0.6, 1.1
Range	(0.0–2.1)	(0.1–1.8)	(0.0–2.1)

*Abbreviations*: *logMAR* Logarithm of the minimum angle of resolution

### Statistical analysis

Continuous factors were summarized using mean and standard deviation, categorical and factors were summarized using percentage. Due to the right skewed distribution of CDE, CDE was first transformed as log10 (CDE+ 1) to normalize its distribution, and this log-transformed measure was used for statistical analysis. Univariable and multivariable linear regression models were used to identify factors associated with CDE. Univariable and multivariable logistic regression models were used to identify factors associated with good postoperative BCVA (i.e., logMAR ≤0.3) at 1 month. In the multivariable analyses for CDE and BCVA, the initial multivariable regression model included all the factors with *p* ≤ 0.20 from the univariable analysis, the model then went through the backward variable selection by keeping the factors with *p* ≤ 0.05 in the final multivariate model. All statistical analyses were performed using the Statistical Analysis Software (SAS version 9.4, North Carolina, USA).

## Results

### Demographics

There were 1294 cases screened for this study. After excluding 64 cases due to missing CDE and 128 cases due to cataract surgery combined with other ocular surgical procedures, a total of 1102 cases (864 cases from UCSF and 238 cases from ZOC) with phacoemulsification were included into analysis. Table 1 summarizes the demographics and clinical characteristics of the study subjects. The mean age (± standard deviation) of patients was 70.7 ± 10.6 years, and 39.6% were male. Regarding medical and ocular history, 23.4% were diabetic, 0.7% had comorbid uveitis, 0.6% had corneal scars or transplants, 24.4% had glaucoma, and 5.8% had retinal pathologies or history of prior retinal procedures. In terms of patient ocular geometry parameters, anterior chamber depth was 3.1 ± 0.5 mm, and axial length was 24.1 ± 1.7 mm. Regarding cataract grading, the majority of them were clinical nuclear sclerotic (NS) cataract grade 2 (47.2%), the average clinical cortical cataract grade was 0.86, and the average posterior subcapsular cataract grade was 0.64. Complications during surgery included 0.5% cases with capsular rupture without vitrectomy, 0.9% cases had capsular rupture with vitrectomy, 0.1% cases with zonular dialysis requiring vitrectomy, and 0.3% cases with other unspecified complications. Attendings or trainees performed the surgeries at UCSF, while attendings performed all surgeries at ZOC. The trainees at UCSF included 2nd year residents, early 3rd year residents who were within the first 6 months of their 3rd year, late 3rd year residents who were in the second 6 months of their 3rd year, and fellows trained for either glaucoma or cornea. All trainee-performed phacoemulsifications were under direct supervision of attending surgeons.

### Factors associated with cumulative dissipated energy

The results from the univariable analysis are reported in Supp. Table 1. In the multivariable analysis, patient age at time of surgery, diabetes, white-to-white corneal diameter, clinical NS grade, preoperative BCVA, cataract disassembly method, surgeon training level, and surgical center were significantly associated with CDE levels (Table 2). Older age and diabetes were associated with higher CDE (increase in log_10_CDE by 0.20–0.31 for age ≥ 70 years, *p* < 0.001; increase in log_10_CDE by 0.07 with diabetes, *p* = 0.007); higher NS grade and greater white-to-white corneal diameter were significantly associated with higher CDE (increase in log_10_CDE by 0.12–0.41 for NS grade ≥ 2, *p* < 0.001; increase in log_10_CDE by 0.48 per 10 mm increase in white-to-white corneal diameter, *p* = 0.008); worse preoperative BCVA was associated with higher CDE (increase in log_10_CDE = 0.16 per logMAR unit increase, *p* < 0.001); use of a disassembly method other than non-stop chop was associated with higher CDE with an increase in log_10_CDE of 0.34 for stop and chop method and 0.47 for other methods. CDE was also higher at UCSF than ZOC by 0.33 log10 CDE (*p* < 0.001). CDE levels were significantly lower when surgery was performed by attendings, fellows, or third year residents compared to second year residents (decrease in log_10_CDE by > 0.09, *p* = 0.03).

**Table 2:** Multivariable Analysis of Factors Associated with Cumulative Dissipated Energy

Characteristics		Combined (***N*** = 1082 eyes)				
		N of eyes	Log10 (CDE+ 1) Adjusted Mean (SE)	Adjusted Difference (95%CI)	*P*-value	Overall P-value
**Patient-Level Characteristics**						
**Age at the time of surgery**	< 60	132	0.70 (0.03)	REF		< 0.001
	60–69	340	0.78 (0.02)	0.08 (0.01, 0.16)	0.03	
	70–79	420	0.90 (0.02)	0.20 (0.12, 0.27)	< 0.001	
	80–89	162	0.99 (0.03)	0.29 (0.20, 0.38)	< 0.001	
	> = 90	28	1.01 (0.08)	0.31 (0.13, 0.48)	< 0.001	
**Diabetics**	No	828	0.84 (0.01)	REF		0.007
	Yes	254	0.91 (0.02)	0.07 (0.02, 0.12)	0.007	
**Eye-Level Characteristics**						
**Nuclear sclerotic cataract grade**	0	21	0.68 (0.06)	REF		< 0.001
	1	173	0.72 (0.03)	0.04 (−0.08, 0.16)	0.56	
	2	516	0.81 (0.02)	0.12 (0.00, 0.24)	0.046	
	3	327	0.97 (0.02)	0.29 (0.17, 0.41)	< 0.001	
	4	45	1.10 (0.05)	0.41 (0.26, 0.57)	< 0.001	
**White-to-white corneal diameter**	Every 10 mm increase	1082		0.48 (0.13, 0.83)		0.008
**Pre-surgery logMAR visual acuity**	Every 1 unit increase	1082		0.16 (0.11, 0.22)		< 0.001
**Surgery-Level Characteristics**						
**Surgeon training level**	Second year resident	2	0.97 (0.06)	REF		0.03
	Early third year resident	190	0.85 (0.02)	−0.12 (−0.24, −0.00)	0.04	
	Late third year resident	234	0.82 (0.02)	−0.16 (− 0.27, − 0.04)	0.006	
	Fellow	242	0.85 (0.03)	−0.13 (− 0.25, − 0.00)	0.04	
	Attending	414	0.88 (0.02)	−0.09 (− 0.21, 0.02)	0.02	
**Disassembly method**	Stop and chop	360	1.07 (0.03)	REF		< 0.001
	Non-stop chop	693	0.73 (0.02)	−0.34 (−0.41, − 0.27)	< 0.001	
	Other	29	1.19 (0.06)	0.13 (0.00, 0.26)	0.048	
**Center**	UCSF	851	0.93 (0.02)	REF		< 0.001
	ZOC	231	0.59 (0.04)	−0.33 (−0.43, −0.24)	< 0.001	

### Factors associated with postoperative visual acuity outcome

Results from the univariable analysis for factors associated with good VA outcome (20/40 or better) were shown in (Suppl. Table 2). In the multivariable analysis, age above 90 years old, retinal disease, axial length (AL), anterior chamber depth (ACD), and preoperative BCVA were significantly associated with postoperative visual acuity outcome (Table 3). Patients older than 90 years had lower odds of good postoperative BCVA compared to patients younger than 60 years (OR = 0.12, *p* < 0.001). Patients with comorbid retinal issues had lower odds of good vision (OR = 0.39, *p* < 0.01). Greater AL was associated with lower odds of good postoperative BCVA (OR = 0.84 per 1 mm increase AL); greater ACD increased the odds of good postoperative outcome (OR = 2.64 per 1 mm increase ACD); worse baseline visual acuity was associated with lower odds of good postoperative BCVA (OR = 0.26 per unit increase in preoperative BCVA logMAR, *p* < 0.001). Visual acuity outcome was not significantly different between the two surgical centers. Notably, NS grade, cataract disassembly method, surgeon training level, and CDE level were not significantly associated with good visual acuity outcome.

**Table 3:** Multivariable Analysis of Factors Associated with Post-surgery Good Vision (VA ≤0.3 logMAR)

Characteristics		Combined (***N*** = 1079 eyes)				
		N of eyes	n(%) of good vision	Adjusted Odds ratioa (95%CI)	*P*-value	Overall P-value
**Age at the time of surgery**	< 60	132	101 (76.5%)	REF		0.002
	60–69	340	280 (82.4%)	1.05 (0.53, 2.08)	0.90	
	70–79	418	333 (79.7%)	0.72 (0.36, 1.43)	0.34	
	80–89	161	124 (77.0%)	0.52 (0.24, 1.13)	0.10	
	> = 90	28	7 (25.0%)	0.12 (0.03, 0.43)	< 0.001	
**Retina comorbid**	None	1016	822 (80.9%)	REF		< 0.001
	Pars plana vitrectomy	27	12 (44.4%)	0.39 (0.18, 0.88)	0.001	
	Other	36	11 (30.6%)	0.13 (0.05, 0.33)	0.02	
**Axial length**	Every 1 mm increase	1079	845 (78.3%)	0.84 (0.76, 0.93)		< 0.001
**Anterior chamber depth**	Every 1 mm increase	1079	845 (78.3%)	2.64 (1.75, 3.97)		< 0.001
**Pre-surgery logMAR visual acuity**	Every 1 unit increase	1079	845 (78.3%)	0.26 (0.19, 0.35)		< 0.001

^a^Odds ratio was defined as the odds of good post-surgery visual outcome with logMAR BCVA score ≤ 0.3

## Conclusion and discussion

In this study of over 1000 cases of phacoemulsification at two independent surgical centers, we tested a broad set of factors for association with intraoperative CDE levels and postoperative BCVA. The motivation for our study is to provide surgeons with reliable risk factor charts including factors that can be practically obtained clinically, to better predict the levels of ultrasound emitted during phacoemulsification, and to better predict the visual acuity outcome when cases are performed with or without trainees. This study provides important information to determine which cataract cases are appropriate for trainees to guide teaching phacoemulsification to residents.

Although the impact of ultrasonic exposure in causing iatrogenic injury of ocular tissue remain debated, current consensus among cataract surgeons is to limit ultrasonic exposure to the ocular tissue during the surgery. Some studies have shown positive correlation between CDE levels and the rate of endothelial cell loss, while others have shown no significant correlation [6–8, 10, 11]. The long-term effect of CDE on cornea condition needs to be further studied. We postulated that while CDE level may not be relevant in determining outcomes of routine cases, it may be important for patients at higher risk for complications. Predicting CDE before cataract surgery for older patients or patients with history of ocular disease might help guide the best course of action to protect corneal tissue.

We found that patient age, clinical NS grade, cataract disassembly method, white-to-white corneal diameter, preoperative BCVA, surgeon training level and surgery center are independent predictors of CDE levels. Comparing within similar cataract gradings, patients of age 70 or older required greater phacoemulsification energy than younger patients, which, to our knowledge, has not been previously reported. This association is clinically important and should be considered when deciding optimal timing for cataract surgery, particularly in eyes with ocular co-morbidities such as corneal disease. We also found that CDE levels were positively correlated with clinical NS grade, which is consistent with the previous reports regarding the relationship between lens density and CDE [12, 13]. This result is particularly noteworthy as it suggests that Scheimpflug images may not be necessary for predicting CDE values during surgery, as cataract density graded under slit lamp exam may serve as a surrogate measure. The association of the non-stop cataract disassembly method with lower CDE values agrees with prior studies [11, 14].

Not surprisingly, we found that second year residents used higher CDE levels than third year residents, fellows or attendings, which is a finding not previously reported. This is consistent with an early trainee’s unfamiliarity with the phacoemulsification system and the need for trainees to develop dexterity over time. Importantly, our findings can be taken into account when deciding which phacoemulsification cases are appropriate for teaching.. For example, early trainees may consider treating younger patients and those with less dense cataracts. Our analysis revealed that CDE levels were different across the two centers. Inclusion of trainees at UCSF is a possible reason. In addition, the difference could be also explained by procedural differences. This effect may be investigated in future dedicated studies comparing data across multiple centers.

Regarding visual acuity outcome at 1 month postoperatively, we found that it was significantly associated with preoperative BCVA, AL, and ACD. Notably, while NS grade, cataract disassembly method, surgeon training level and surgical center were associated with CDE levels, these factors were not associated with postoperative BCVA. CDE levels alone were also not associated with postoperative BCVA. Together, these results suggest that while CDE levels are heterogenous across cases and can be higher when surgery is performed by supervised residents in their early training stages, this variation may have no consequence on surgical outcome as quantified by postoperative BCVA, when direct supervision is provided. It should be noted that the visual acuity was measured 1 month postoperatively, and tissue damage that may occur during surgery may take months or years to manifest clinically. Future studies will be necessary to fully understand the factors determining cataract surgery outcome in the long-term.

A limitation of the study is that there was no trainee data at ZOC to compare with that at UCSF. Secondly, ZOC and UCSF have disparate patient populations. However, this may help generalize our results to different ethnicities and surgical settings. Thirdly, some phacoemulsification parameters, such as phacoemulsification time, were not included in this large cohort study. However, because CDE integrates the amount of ultrasonic energy delivered to the eye over the course of the procedure, there is a structural positive association between effective phacoemulsification time and CDE. Thus, effective phacoemulsification time is implicitly embedded in this analysis through CDE, and our results that CDE is not significantly associated with postoperative BCVA are consistent with these prior studies reporting that effective phacoemulsification time is not associated with postoperative outcome [15]. Lastly, because of the low rate of complications, we were not able to analyze the association between complications and CDE postoperative visual acuity.

Overall, our series of cases from two independent institutions reveals that CDE levels and postoperative BCVA are affected by a variety of factors. Clinically determined NS grade under slit lamp, age of surgery, and resident at early training stage are newly identified risk factors correlated with CDE levels. BCVA at 1 month postoperatively is uncorrelated with CDE levels during cataract surgery. Greater experience of the primary surgeon was associated with lower CDE levels. However, our study indicates that in terms of visual acuity outcome, phacoemulsification is as safe and effective when performed by supervised residents as when performed by attendings.

None of the authors have any conflicts of interest or financial disclosures.

## Supplementary Information

**Additional file 1.**

## Data Availability

The datasets used and/or analyzed during the current study are available from the corresponding author on reasonable request.
