# Supplementary material for: Factors impacting cumulative dissipated energy levels and postoperative visual acuity outcome in cataract surgery
Source: BMC Ophthalmol. 2021 Dec 20;21:439. doi: 10.1186/s12886-021-02205-w (PMC8690865; doi:10.1186/s12886-021-02205-w)
Supplement: Supplementary file 1 — Additional file 1. [file 12886_2021_2205_MOESM1_ESM.docx]

**Factors Impacting Cumulative Dissipated Energy Levels and Postoperative Visual Acuity Outcome in Cataract Surgery**

*Anh D. Bui, MD, PhD^1^,^1,*^ Zhiming Sun,^2,*^ Yunzhen Wang, ^2,*^ Shengsong Huang, ^2^ Michael Ryan, MD,^1^ Yinxi Yu, MS,^3^ Gui-Shuang Ying, MD, PhD,^3^ Saras Ramanathan, MD,^1^ Kuldev Singh, MD, MPH,^4^ Yangfan Yang, MD, PhD, ^2,#^ Ying Han, MD, PhD^1,#^*

Table S1 Univariable Analysis of Factors Associated with Cumulative Dissipated Energy

|  |  | **Combined (N=1102 eyes)** | | | | |
| --- | --- | --- | --- | --- | --- | --- |
| Characteristics |  | N of eyes | Log10 (CDE+1) Mean (SE) | Unadjusted Difference (95%CI) | P-value | Overall P-value |
| **Patient-Level Characteristics** | | | | | | |
| **Age at the time of surgery** | <60 | 137 | 0.69 (0.04) | REF |  | <0.001 |
|  | 60-69 | 349 | 0.78 (0.02) | 0.09 (0.00, 0.18) | 0.04 |  |
|  | 70-79 | 424 | 0.88 (0.02) | 0.19 (0.10, 0.27) | <0.001 |  |
|  | 80-89 | 163 | 1.02 (0.03) | 0.33 (0.23, 0.43) | <0.001 |  |
|  | >=90 | 29 | 1.16 (0.08) | 0.46 (0.29, 0.64) | <0.001 |  |
| **Gender** | Male | 436 | 0.88 (0.02) | REF |  | 0.10 |
|  | Female | 666 | 0.84 (0.02) | -0.04 (-0.09, 0.01) | 0.10 |  |
| **Diabetics** | No | 844 | 0.84 (0.01) | REF |  | 0.03 |
|  | Yes | 258 | 0.90 (0.02) | 0.07 (0.01, 0.13) | 0.03 |  |
| **Eye-Level Characteristics** | | | | | | |
| **Uveitis comorbid** | No | 1094 | 0.85 (0.01) | REF |  | 0.73 |
|  | Yes | 8 | 0.93 (0.21) | 0.08 (-0.38, 0.54) | 0.73 |  |
| **Cornea comorbid** | No | 1096 | 0.85 (0.01) | REF |  | 0.38 |
|  | Yes | 6 | 1.02 (0.21) | 0.17 (-0.21, 0.55) | 0.38 |  |
| **Glaucoma comorbid** | No | 834 | 0.86 (0.01) | REF |  | 0.14 |
|  | Incisional surgery | 24 | 0.96 (0.08) | 0.11 (-0.03, 0.24) | 0.12 |  |
|  | Pseudoexfoliation glaucoma | 24 | 1.04 (0.11) | 0.18 (-0.05, 0.42) | 0.13 |  |
|  | Primary angle closure disease | 57 | 0.82 (0.05) | -0.04 (-0.14, 0.06) | 0.47 |  |
|  | Other | 163 | 0.81 (0.03) | -0.05 (-0.12, 0.03) | 0.24 |  |
| **Retina comorbid** | None | 1039 | 0.84 (0.01) | REF |  | 0.001 |
|  | Pars plana vitrectomy | 27 | 1.06 (0.07) | 0.21 (0.07, 0.35) | 0.003 |  |
|  | Other | 36 | 0.97 (0.05) | 0.12 (0.02, 0.22) | 0.02 |  |
| **Nuclear sclerotic cataract grade** | 0 | 23 | 0.50 (0.06) | REF |  | <0.001 |
|  | 1 | 177 | 0.64 (0.03) | 0.15 (0.02, 0.28) | 0.03 |  |
|  | 2 | 520 | 0.81 (0.02) | 0.31 (0.19, 0.43) | <0.001 |  |
|  | 3 | 331 | 1.02 (0.02) | 0.53 (0.40, 0.65) | <0.001 |  |
|  | 4 | 45 | 1.18 (0.04) | 0.69 (0.54, 0.83) | <0.001 |  |
| **Cortical cataract grade** | 0 | 578 | 0.83 (0.02) | REF |  | 0.07 |
|  | 1 | 206 | 0.90 (0.02) | 0.07 (0.00, 0.13) | 0.04 |  |
|  | 2 | 208 | 0.89 (0.03) | 0.05 (-0.01, 0.12) | 0.12 |  |
|  | 3 | 99 | 0.80 (0.03) | -0.04 (-0.11, 0.04) | 0.32 |  |
|  | 4 | 8 | 0.84 (0.17) | 0.00 (-0.30, 0.31) | 0.98 |  |
| **Posterior subcapsular cataract grade** | 0 | 721 | 0.84 (0.01) | REF |  | 0.049 |
|  | 1 | 191 | 0.88 (0.03) | 0.04 (-0.02, 0.10) | 0.23 |  |
|  | 2 | 82 | 0.85 (0.04) | 0.01 (-0.07, 0.10) | 0.74 |  |
|  | 3 | 70 | 0.88 (0.05) | 0.04 (-0.07, 0.14) | 0.47 |  |
|  | 4 | 34 | 0.99 (0.05) | 0.15 (0.05, 0.25) | 0.003 |  |
| **Axial length** | Every 10 mm increase | 1091 |  | 0.02 (-0.14, 0.18) |  | 0.80 |
| **Anterior chamber depth** | Every 10 mm increase | 1077 |  | -0.25 (-0.66, 0.17) |  | 0.24 |
| **White-to-white corneal diameter** | Every 10 mm increase | 1089 |  | 0.26 (-0.13, 0.65) |  | 0.19 |
| **Pre-surgery logMAR visual acuity** | Every 1 unit increase | 1098 |  | 0.22 (0.17, 0.27) |  | <0.001 |
| **Surgery-Level Characteristics** | | | | | | |
| **Surgeon training level** | Second year resident | 2 | 1.17 (0.00) | REF |  | <0.001 |
|  | Early third year resident | 192 | 0.86 (0.03) | -0.31 (-0.37, -0.25) | <0.001 |  |
|  | Late third year resident | 236 | 0.77 (0.02) | -0.40 (-0.45, -0.35) | <0.001 |  |
|  | Fellow | 246 | 0.86 (0.03) | -0.30 (-0.37, -0.24) | <0.001 |  |
|  | Attending | 426 | 0.89 (0.02) | -0.28 (-0.31, -0.24) | <0.001 |  |
| **Disassembly method** | Stop and chop | 367 | 1.02 (0.02) | REF |  | <0.001 |
|  | Non-stop chop | 705 | 0.75 (0.01) | -0.27 (-0.32, -0.22) | <0.001 |  |
|  | Other | 29 | 1.30 (0.06) | 0.28 (0.15, 0.40) | <0.001 |  |
| **Pupil expansion or capsular support devices** | No | 1071 | 0.85 (0.01) | REF |  | 0.20 |
|  | Yes | 31 | 0.94 (0.07) | 0.09 (-0.05, 0.22) | 0.20 |  |
| **Loose zonules** | No | 1090 | 0.85 (0.01) | REF |  | 0.01 |
|  | Yes | 12 | 1.15 (0.13) | 0.30 (0.06, 0.54) | 0.01 |  |
| **Complication status** | No | 1081 | 0.85 (0.01) | REF |  | 0.33 |
|  | Yes | 21 | 0.94 (0.09) | 0.09 (-0.09, 0.27) | 0.33 |  |
| **Center** | UCSF | 864 | 0.82 (0.01 | REF |  | <0.001 |
|  | ZOC | 238 | 0.96 (0.02) | 0.13 (0.08, 0.19) | <0.001 |  |

Table S2 Univariable Analysis of Factors Associated with Post-surgery Good Vision

|  |  | **Combined (N=1102 eyes)** | | | | |
| --- | --- | --- | --- | --- | --- | --- |
| Characteristics |  | N of eyes | n(%) of good vision | Unadjusted Odds ratio (95%CI) | P-value | Overall P-value |
| **Patient-Level Characteristics** | | | | | | |
| **Age at the time of surgery** | <60 | 137 | 106 (77.4%) | REF |  | <0.001 |
|  | 60-69 | 349 | 287 (82.2%) | 1.35 (0.80, 2.30) | 0.26 |  |
|  | 70-79 | 424 | 336 (79.2%) | 1.12 (0.67, 1.86) | 0.67 |  |
|  | 80-89 | 163 | 126 (77.3%) | 1.00 (0.55, 1.82) | 0.99 |  |
|  | >=90 | 29 | 8 (27.6%) | 0.11 (0.04, 0.33) | <0.001 |  |
| **Gender** | Male | 436 | 347 (79.6%) | REF |  | 0.44 |
|  | Female | 666 | 516 (77.5%) | 0.88 (0.64, 1.21) | 0.44 |  |
| **Diabetics** | No | 844 | 652 (77.3%) | REF |  | 0.16 |
|  | Yes | 258 | 211 (81.8%) | 1.32 (0.89, 1.95) | 0.16 |  |
| **Eye-Level Characteristics** | | | | | | |
| **Uveitis comorbid** | No | 1094 | 858 (78.4%) | REF |  | 0.32 |
|  | Yes | 8 | 5 (62.5%) | 0.46 (0.10, 2.14) | 0.32 |  |
| **Cornea comorbid** | No | 1096 | 861 (78.6%) | REF |  | 0.02 |
|  | Yes | 6 | 2 (33.3%) | 0.14 (0.02, 0.75) | 0.02 |  |
| **Glaucoma comorbid** | No | 834 | 662 (79.4%) | REF |  | 0.14 |
|  | Incisional surgery | 24 | 14 (58.3%) | 0.36 (0.14, 0.91) | 0.03 |  |
|  | Pseudoexfoliation glaucoma | 24 | 16 (66.7%) | 0.52 (0.21, 1.30) | 0.16 |  |
|  | Primary angle closure disease | 57 | 42 (73.7%) | 0.73 (0.37, 1.45) | 0.36 |  |
|  | Other | 163 | 129 (79.1%) | 0.99 (0.63, 1.55) | 0.95 |  |
| **Retina comorbid** | None | 1039 | 840 (80.8%) | REF |  | <0.001 |
|  | Pars plana vitrectomy | 27 | 12 (44.4%) | 0.19 (0.09, 0.41) | <0.001 |  |
|  | Other | 36 | 11 (30.6%) | 0.10 (0.05, 0.21) | <0.001 |  |
| **Nuclear sclerotic cataract grade** | 0 | 23 | 21 (91.3%) | REF |  | <0.001 |
|  | 1 | 177 | 155 (87.6%) | 0.67 (0.15, 3.06) | 0.61 |  |
|  | 2 | 520 | 419 (80.6%) | 0.40 (0.09, 1.68) | 0.21 |  |
|  | 3 | 331 | 241 (72.8%) | 0.26 (0.06, 1.09) | 0.07 |  |
|  | 4 | 45 | 22 (48.9%) | 0.09 (0.02, 0.43) | 0.002 |  |
| **Cortical cataract grade** | 0 | 578 | 469 (81.1%) | REF |  | 0.04 |
|  | 1 | 206 | 161 (78.2%) | 0.83 (0.55, 1.26) | 0.38 |  |
|  | 2 | 208 | 160 (76.9%) | 0.77 (0.52, 1.16) | 0.22 |  |
|  | 3 | 99 | 67 (67.7%) | 0.49 (0.29, 0.81) | 0.005 |  |
|  | 4 | 8 | 4 (50.0%) | 0.23 (0.04, 1.29) | 0.09 |  |
| **Posterior subcapsular cataract grade** | 0 | 721 | 577 (80.0%) | REF |  | 0.15 |
|  | 1 | 191 | 148 (77.5%) | 0.86 (0.58, 1.27) | 0.45 |  |
|  | 2 | 82 | 57 (69.5%) | 0.57 (0.34, 0.96) | 0.03 |  |
|  | 3 | 70 | 55 (78.6%) | 0.92 (0.50, 1.69) | 0.78 |  |
|  | 4 | 34 | 23 (67.6%) | 0.52 (0.25, 1.10) | 0.09 |  |
| **Axial length** | Every 1 mm increase | 1094 |  | 0.93 (0.85, 1.02) |  | 0.10 |
| **Anterior chamber depth** | Every 1 mm increase | 1080 |  | 2.02 (1.44, 2.83) |  | <0.001 |
| **White-to-white corneal diameter** | Every 1 mm increase | 1092 |  | 1.20 (0.93, 1.54) |  | 0.16 |
| **Pre-surgery logMAR visual acuity** | Every 1 unit increase | 1102 |  | 0.26 (0.20, 0.33) |  | <0.001 |
| **Surgery-Level Characteristics** | | | | | | |
| **Surgeon training level** | Second year resident | 2 | 1 (50.0%) | REF |  | 0.06 |
|  | Early third year resident | 192 | 158 (82.3%) | 4.65 (0.28, 76.37) | 0.28 |  |
|  | Late third year resident | 236 | 194 (82.2%) | 4.62 (0.28, 75.42) | 0.28 |  |
|  | Fellow | 246 | 195 (79.3%) | 3.82 (0.23, 62.35) | 0.35 |  |
|  | Attending | 426 | 315 (73.9%) | 2.84 (0.18, 45.78) | 0.46 |  |
| **Disassembly method** | Stop and chop | 367 | 267 (72.8%) | REF |  | 0.002 |
|  | Non-stop chop | 705 | 576 (81.7%) | 1.67 (1.23, 2.27) | 0.001 |  |
|  | Other | 29 | 19 (65.5%) | 0.71 (0.31, 1.66) | 0.43 |  |
| **Pupil expansion or capsular support devices** | No | 1071 | 841 (78.5%) | REF |  | 0.36 |
|  | Yes | 31 | 22 (71.0%) | 0.67 (0.28, 1.57) | 0.36 |  |
| **Loose zonules** | No | 1090 | 856 (78.5%) | REF |  | 0.10 |
|  | Yes | 12 | 7 (58.3%) | 0.38 (0.12, 1.22) | 0.10 |  |
| **Complication status** | No | 1081 | 846 (78.3%) | REF |  | 0.77 |
|  | Yes | 21 | 17 (81.0%) | 1.18 (0.39, 3.55) | 0.77 |  |
| **Center** | UCSF | 864 | 698 (80.8%) | REF |  | <0.001 |
|  | ZOC | 238 | 165 (69.3%) | 0.54 (0.38, 0.75) | <0.001 |  |
| **CDE** | Every 1 unit increase | 1098 |  | 0.98 (0.97, 0.99) |  | 0.007 |
